# Use of complementary and alternative medicines by a sample of Turkish women for infertility enhancement: a descriptive study

**DOI:** 10.1186/1472-6882-10-11

**Published:** 2010-03-22

**Authors:** Tamer Edirne, Secil Gunher Arica, Sebahat Gucuk, Recep Yildizhan, Ali Kolusari, Ertan Adali, Muhammet Can

**Affiliations:** 1 Department of Family Medicine, University of Yuzuncu Yil, Van, Turkey; 2 Mother-Child Health and Family Planning Center, Van, Turkey; 3 Department of Gynecology and Obstetrics, University of Yuzuncu Yil, Van, Turkey; 4 Department of Forensic Medicine, University of Yuzuncu Yil, Van, Turkey

## Abstract

**Background:**

Infertility patients are a vulnerable group that often seeks a non-medical solution for their failure to conceive. World-wide, women use CAM for productive health, but only a limited number of studies report on CAM use to enhance fertility. Little is known about traditional and religious forms of therapies that are used in relation to conventional medicine in Turkey. We investigated the prevalence and types of complementary and alternative medicine (CAM) used by infertile Turkish women for fertility enhancement.

**Methods:**

A face-to-face questionnaire inquiring demographic information and types of CAM used for fertility enhancement were completed by hundred infertility patients admitted to a primary care family planning centre in Van, Turkey between January and July 2009.

**Results:**

The vast majority of infertile women had used CAM at least once for infertility. CAM use included religious interventions, herbal products and recommendations of traditional "hodja's" (faith healers). Of these women, 87.8% were abused in the last 12 months, 36.6% felt not being supported by her partner and 80.5% had never spoken with a physician about CAM.

**Conclusions:**

Infertile Turkish women use complementary medicine frequently for fertility enhancement and are in need of information about CAM. Religious and traditional therapies are used as an adjunct to, rather than a substitute for, conventional medical therapy. Physicians need to approach fertility patients with sensitivity and should be able to council their patients about CAM accordingly.

## Background

According to the World Health Organization (WHO), more than three-quarters of the world's population rely upon complementary and alternative medicine (CAM) for health care. The Cochrane Collaboration's definition of CAM is "a broad domain of healing resources that encompasses all health systems, modalities, and practices and their accompanying theories and beliefs other than those intrinsic to the politically dominant health systems of a particular society or culture in a given historical period". There has been a rise in the use of CAM as a health care option in recent years globally [1-4]. In the United States consumers spend over $34 billion per year on CAM therapies and made 354 million visits to CAM practitioners [5]. Australians spend AUD$85 million on consultations with naturopaths and herbalists in the year 2004 [6]. We don't know how much is spent on CAM in Turkey but there are increasing data about CAM use nowadays. The factors influencing CAM use may differ from country to country. Several factors are found to be associated with CAM use in developed countries: dissatisfaction with outcomes associated with conventional medicine [7,8]; a need for more control in healthcare decisions [9-11]; treatment of chronic illnesses [11,12]; and the apparent acceptance of naturalness and harmlessness of CAM [13,14]. In addition, cultural beliefs and practices often lead to self-care, home remedies or consultation with traditional and religious healers in developing or Islamic countries [15-17].

CAM are commonly used in conjunction with conventional medical treatments, however information about CAM use is generally not inquired by doctors nor provided by them to their patients [18-21].

It is assumed that infertility affects 15% of Turkish couples and an increasing number of couples seek assisted reproductive technologies (ART) to achieve parenthood. The pressure to conceive means that some women become 'desperate' to try anything to conceive. World-wide, women use CAM fore productive health, but only a limited number of studies report on CAM use to enhance fertility [22-24]. While the types of complementary therapies used during pregnancy have been documented, there has been little research exploring CAM treatments for infertility and details about the type of therapies and how they are used in relation to conventional medicine are scarce [25,26]. Further research into women's use of CAM for reproductive health is suggested to ensure that doctors are aware that a growing proportion of women who consult them may be using CAM [27].

We could not find any study in Turkey that explored the use of CAM during pregnancy or for the treatment of infertility. However, various complementary therapies, including spiritual are well known among the community in Turkey [28,29], mostly used by adult and child cancer patients [21,30].

The current study was planned to investigate CAM use among infertile Turkish women for the enhancement of fertility. The objective was to find out which nonmedical treatments are being used by infertile women seeking assisted reproduction treatment, and to determine accompanying characteristics.

## Methods

Data was gathered from women seeking knowledge about infertility treatment at a family planning centre in Van, Turkey. The Centers for Mother-Child Health and Family Planning (CFP) in Turkey offer routine care for women and children. In general, one centre exists in a city displaying a supervisory role for the health personnel from the other primary health care centers through in-service educational activities. The CFP in Van operates policlinics including gynecology, family planning counseling, and routine antenatal care. Three family physicians, two primary care physicians, six midwifes give service to the population of Van.

The female population admitted to the center is homogeneous in terms of ethnicity, religion and language. All patients in our sample were born and grown up in this region, were Muslims and all were speaking Kurdish (and some spoke Turkish). According to the data of the local health administrative, a total of 238.582 women aged 15-49 were recorded, with a crude birth rate of 25.9/1000 in the year 2008. There are no data about infertility rates from this region. Including criteria of the women were being 18 years or older, born and living in Van region, and being treated with ovulation induction.

### Sample and survey questionnaire

A face-to-face questionnaire was undertaken with consecutive 100 infertile patients admitted to the Family Planning Centre in Van, Turkey from January to July 2009.

The questionnaire was structured after preliminary discussions with patients and health professionals who were also informed by a literature review. Women, who admitted to the centre seeking information about ovulation induction, were invited to participate.

In the first part, subjects were investigated regarding demographic information that was suspected to influence the patients' health-related behaviors including age, education and income level, years of infertility, gravidity, parity, household structure and locality. Questions inquiring partner support, intimate partner violence (IPV) and knowledge about CAM were also asked. In the second part, respondents were asked to mention therapies they had used expressly for the purpose of getting pregnant.

### Data recording and analysis

All of the interviews were conducted by trained female family physicians. Interviews took place in the visit room individually and lasted approximately 20 minutes. A midwife speaking Kurdish was ready for translation.

Because the study was descriptive by nature, no power analysis was performed in advance. Demographic information was not compared between those using alternative treatments and nonusers because of the insufficient number of nonusers. This study was determined to be exempt from review by the institutional review board at the University of Yuzuncu Yil. All participants gave informed consent.

## Results

A total of 100 women accepted to participate the study out of 115 invited (86.0% response rate). Overall, 82% of the women had used CAM once for infertility. Mean age of these CAM users was 26.7 (range of 18 - 40 years). Mean duration of infertility was 8.0 years (7-months - 27 years). Educational and economical level was low in general. Of the women who had used CAM for infertility treatment, 87.8% was abused in the last 12 months, 36.6% felt not supported by her partner and 80.5% had never spoken with a physician about CAM (Table 1).

**Table 1:** Characteristics of infertile patients who used CAM in Van, Turkey 2009 (n = 82)

Characteristic	
Mean age, years (SD)	26.7 (6.0)
Years of infertility (SD)	8.0 (5.9)
Gravidity (SD)	0.9 (1.2)
Parity (SD)	0.4 (0.9)
	**No (%)**
Educational level and attendance of school in years	
Illiterate	40 (48.8)
< 5 years	32 (39.0)
≥ 5 years	10 (12.2)
Monthly household income	
< Below minimum wage rate (US $377)*	51 (62.2)
≥ Equal or above min. wage rate (US $377)	31 (37.8)
Location	
Rural	55 (67.1)
Urban	27 (32.9)
Living with parents-in-law	57 (69.5)
Abused in the last 12 months (%)	72 (87.8)
Feels not supported by her partner (%)	30 (36.6)
Never spoke with a physician about CAM (%)	66 (80.5)

*Minimum wage rate 2008, Turkey (1 USD = 1.21 TL)

The most common intervention complementary to standard medical therapy was religious intervention used by all respondents. The next most common intervention involved faith healers with 46.3% (38) stating that they sought help from faith healers (hodja's) and accepted to use several folkloric remedies recommended by them. The proportion of patients who used herbals and visited faith healers was 36.6% (30). The use of herbals alone and folkloric methods was reported by 29.3% (24) and 11.0% (9), respectively.

The patterns of CAM used by the patients reflected well-known herbal medicines but local traditional remedies were also reported.

### Herbals

Dried leaves or roots of nettle (*Urtica dioica L*.) are boiled in water and steeped for 3-5 minutes. It is consumed several times a day as a hot (tea) or cold beverage.

European black pine (*Pinus nigra *Arnold) and subspecies *Pinus nigra *ssp. *caramanica *[31] belonging to Pinaceae family grows on Taurus Mountains in Southern Anatolia. Tar (resin) is obtained by dry-distillation from the stem and branches in primitive holes in the ground. It is a semi-solid black liquid, which is externally applied to the para-umblical region of the infertile women in form of a plaster for at least three days.

Fresh leaves of Johnny jumpup (wild daffodil; *Viola tricolour *L.) or heartsease (*Narcissus pseudonarcissus*) are squeezed together to form an ovule like small pill, which are inserted to the vagina before sexual intercourse.

### Folkloric methods

Two spoons of wheat germ oil are swallowed twice every day and the patient sits naked on hot ashes or hot bricks for several days. Some prefer thermal spas with the same purpose.

The reproductive organ (uterus) of the female rabbit (Oryctolagus cuniculus) is cooked and eaten before sexual intercourse.

### Faith healings

Patients may consult to religious healers. The *hodja *has a special spiritual power, acquired through inheritance (lineage) or a lifetime of devotional acts, allows him to communicate directly with God and thus act as a mediator between God and the people. Hodja's offer a number of treatments and practice traditional systems of medicine, which involves use of a variety of herbs and minerals. Amulets (*tawiz*), containing verses from the Koran written by hodja's are usually worn around the neck; act as a defense against evil spirits or the evil eye (*nazaar*). Hodja's may also give cure through their breaths (healing breath) by blowing his breath over the body. He also may blow water or food (rice, for example) and then the blessed water is drunk or the food is eaten.

When the problem is thought to be spiritual, the hodja may diagnose possession by evil spirits (*jinns*), which must be exorcised. However, only specialist *hodja's *have the specific knowledge to perform exorcisms. There is a widespread belief amongst Muslims that *jinns *are spiritual beings - created from smokeless fire rather than the spirit of dead people - that live on earth in a world parallel to mankind. *Jinns *have the ability to possess and take over the minds and bodies of other creatures, including humans, and to behave in either a good or evil manner. *Jinns *possess people for different reasons. Most of the time possession occurs because the *jinn *is simply malicious and wicked.

### Religious healings

The widespread religious health-seeking behaviors involve individuals or groups praying or reciting religious texts to seek cure. Individuals may drink holy water from hajj, fast or undertake pilgrimages to holly places (graves) to seek forgiveness of sins and alleviation of illness. At some holly places, people light candles or bind pieces of their cloths to the trees with a wish to conceive.

Additional file 1: Table S1 summarizes complementary and traditional approaches mentioned above.

## Discussion

This study shows that many infertile Turkish women are using nonmedical treatments and interventions in addition to those used in medical practice. The most prevalent intervention included items summarized as religious healing which can exert positive influences on health by contributing to a sense of hope and allowing coping with the stress of infertility treatment [32]. People having faith in spiritual healers, clergymen, hodja's, homeopaths or even many quacks, have utilized alternative therapies. These are the first choice for problems such as infertility, epilepsy, psychosomatic troubles, depression, etc [33].

Religion has a strong influence on people's beliefs about illness and treatments resulting in acceptance of methods recommended by religious healers (hodja) without rational criticism. In our study, all of our patients were praying for cure in routine and nearly half of them had visited a faith healer (hodja) and complied with his methods, even if they were inconceivable.

Spiritual healing methods are known to be used regularly for relive by Turkish patients [34] and in the Muslim world [35] but also in the western world [36].

However, one might argue this is a true reflection of the patients' culture where prayer and spiritual believes are part of people's everyday life and may not be included in CAM.

Some religious methods included in this study have never been shown to directly affect fertility, but some may indirectly influence health in a negative way. Remedies recommended by hodja such as "holly breath and foods" must be mind confusing. Methods such as exorcism could have a devastating effect on the psychological health of the patients who already are suffering emotional distress. They are based on the false belief that illness is the expression of sins that can be manipulated by some religious interventions. Generally, these interventions are sold to the patients with the promise that they can cure multiple diseases, such as infertility. All are aimed at vulnerable clients desperate for anything that promises hope.

Few herbal supplements have been subjected to adequate study regarding efficacy on fertility. We found stinging nettle to be the most frequently used herbal supplement, and it is recommended for many illnesses including cancer [21,37-40].

A review of literature on nettle, however, does not find adequate support for its use in infertility [41]. Nettle leaf has traditionally been used for numerous other conditions, although confirmatory clinical trials have not been conducted for all remedies. Moreover, although it is believed to be generally safe, it can cause adverse effects by interfering with some drugs.

The use of medicinal tar (resin, pitch) for dermatological disorders dates back to the ancient times. Although coal tar is utilized more frequently in modern dermatology, wood tars have also been widely employed. Wood tars have been used in the treatment of various cutaneous disorders, including psoriasis and atopic dermatitis, and have no photosensitizing effects [42]. An increased risk of irritation and allergic sensitization has been seen with their use [43,44].

No relevant information was found in the literature on the benefits of tar to infertility.

In one scientific study, the ethanol extract of the bulbs of *Narcissus pseudonarcissus *was found effective in one mouse model of nociception, para-benzoquinone induced abdominal constriction, but not in another, the hot plate test. However, at these concentrations it also caused significant toxic effects [45].

*Viola tricolor *is one of many plant species containing cyclotides. These small peptides have proven to be useful in drug development due to their size and structure giving rise to high stability. One such cyclotide, vitri A, found in *Viola tricolor *is said to contain cytotoxic characteristics [46] meaning that it could be used to treat cancers but no scientific study regarding its effects on fertility was found.

For other folkloric interventions stated here, no data exist. The opinion that ingesting organs of highly reproductive animals will make conception more likely could be dating back to old times but will never be studied and are generally only of cultural interest. This is also true for the combination of wheat germ and heating the female genitourinary tract, which may exhibit a response to the cold climate of this region with long and severe winters.

Given that infertility rituals are described more in less developed cultures, cultural and national influences are likely to affect the use of nonmedical treatments. The nonmedical therapies identified by this study are prevalent in this community, and may not be generalized to other cultures and time periods. Still, many of these recommendations have existed for generations, and the fact that they are still in use suggests that some must be useful or effective. Very few, however, have actually been studied for evidence of their efficacy.

Studies from developing, Eastern or pronatalist countries tend to focus on society's stigmatization of infertility [47,48]), the lack of support from husbands [49-52] and the importance of education and counseling about infertility and treatment approaches to infertility [53-55]. Our results support these findings. We only included patients treated by conventional ovulation induction methods into the study. The use of CAM was widespread, although only in means of a second-line rather than an alternative. The majority had never discussed CAM with a doctor which is widespread pattern of patients and even physicians in Turkey are in need of information about CAM [34,56].

Furthermore, we also documented that infertility patients were subject to IPV, which in general originates from husbands. Intimate partner violence is known to be frequent among Turkish couples, and infertile women report high rates [57-59]. We suggest that there is a cultural background underlying these findings.

Living with parents-in-law may create some emotional pressure from mothers' and fathers' in-law, which is well documented all over the world [59-62]. Motherhood is believed to be the most important role for women and is fundamental for the women's identity in Islamic culture. The public places great emphasis on fertility and childbearing, which in turn is a matter of honor for both families. Therefore, decisions to seek alternative treatments are to be usually taken as a family rather than as an individual and people may having been coerced or persuaded into accepting them, as it has been reported before [63].

Attributing the causes of infertility to supernatural causes such as evil spirits and God's retribution, and seeking help from faith and traditional healers may be a projection of the social stigmatization for infertile women and the strong desire for motherhood. This desire was described by infertile women as a willingness to try almost anything to maximize their chances of becoming pregnant, reflected in the variety of CAM modalities they invested in and their use of ART [64].

The prevalence of CAM use in this sample of infertile Turkish women indicates the appropriateness of counseling these patients about CAM through evidence based knowledge. Successful programs in dealing with infertility need to include the establishment of a community based intervention strategy including primary care physicians to educate people about infertility and to give guidelines for treatment options. Because of the popularity of these non-prescription treatment methods, it is important for healthcare providers to be prepared to initiate discussions with their patients and provide counseling.

This study has several limitations including the collection of data by means of the questionnaire, which introduced the threat of selection bias. The validity of the findings is dependent on the individual's memory and accuracy in reporting CAM use. The survey methodology also carries an inherent selection bias. A relatively small fraction of patients in one health facility chose to complete the survey. Women who have an interest in complementary and alternative medicines might have been more likely to take the time to complete the questionnaire. The sample in this study reflects only one area of Turkey and the findings should be limited to this population. Nevertheless, this is the first study on CAM use in fertility patients in Turkey.

## Conclusions

For the most part, nonmedical treatments in this study were irrational and possibly dangerous. Although religious interventions are of little proven benefit, they may contribute positively to infertility treatment either by giving a sense of empowerment or control or by helping to relieve some of the stress. On the other hand, some interventions may involve substantial emotional distress, such as exorcism, with no known benefit. Some herbal preparations may even have an ill effect on health and well-being. It is important that health professionals are aware of their patients' lay beliefs about illness and the CAM that they may choose. Medical personnel need to take into account the religious dimensions of the experience of infertility while caring for patients. Physicians providing care for infertile patients may therefore should inquire about such nonmedical practices and be able to counsel their patients accordingly.

## Competing interests

The authors declare that they have no competing interests.

## Authors' contributions

TE participated in the design of the study, acquisition and interpretation of data, helped to draft the questionnaire, and participated in writing the discussion and revised it. SGA participated in the design of the study, helped to draft the questionnaire and to discuss the results. SG conceived of the study, and participated in its design and coordination and helped to draft the manuscript. AK discussed and evaluated the results and corrected the manuscript for publication. RY helped draft the manuscript, discussed the results and participated in the revision of the study. EA evaluated the results, participated in the discussions and in the revision of the study. MC participated in the design and survey and in the revision of the study. All authors read and approved the final manuscript.

## Pre-publication history

The pre-publication history for this paper can be accessed here:

http://www.biomedcentral.com/1472-6882/10/11/prepub

## Supplementary Material

Additional file 1 **Table S1**. Non-medical treatments used for infertility in the region of Van as reported by the participants, Turkey 2009. Click here for file
