# Supplementary material for: Use of complementary and alternative medicines by a sample of Turkish women for infertility enhancement: a descriptive study
Source: BMC Complement Altern Med. 2010 Mar 22;10:11. doi: 10.1186/1472-6882-10-11 (PMC2853488; doi:10.1186/1472-6882-10-11)
Supplement: Additional file 1 — Table S1. Non-medical treatments used for infertility in the region of Van as reported by the participants, Turkey 2009. [file 1472-6882-10-11-S1.DOC]

**Additional file 1: Table S1.** Nonmedical treatments used for infertility in the region of Van as reported by the participants, Turkey 2009

| ***Herbals*** |
| --- |
| Stinging nettle |
| Tar (resin) |
| Johnny jumpup (Viola tricolor) leaves |
| Heartsease (Narcissus pseudonarcissus) leaves |
| ***Folkloric methods*** |
| Hot ashes or hot bricks |
| Rabbit sex organs |
| ***Faith healing*** |
| Holly amulets, water or food |
| Exorcism |
| ***Religious healing*** |
| Praying |
| Pilgrimage |
